# Developing a biostatistical support system in a resource-restricted academic institution in Africa: making it happen

**DOI:** 10.1186/s12909-015-0493-0

**Published:** 2015-11-25

**Authors:** Tobias Chirwa, Beverley Kramer, Elena Libhaber

**Affiliations:** 1 Division of Epidemiology and Biostatistics, School of Public Health, Faculty of Health Sciences, University of the Witwatersrand, Johannesburg, Republic of South Africa; 2 Health Sciences Research Office, Faculty of Health Sciences, University of the Witwatersrand, Johannesburg, Republic of South Africa; 3 School of Clinical Medicine, Faculty of Health Sciences, University of the Witwatersrand, Johannesburg, Republic of South Africa

## Abstract

**Background:**

In order to address and support biostatistics for health research, the Health Sciences Research Office of the University of the Witwatersrand sought to introduce training in biomedical statistics to sustain research and postgraduate education. The experiences encountered in setting up such statistical support in a limited resource, developing country are discussed here.

**Methods:**

Two cross-sectional surveys (a) statistical needs assessment (2009) and (b) feedback (2010–11) on the statistical support through biostatistics courses and consultations were conducted. These surveys were supplemented with information such as graduations, research publication output and costs of setting up the support.

**Results:**

Seventy-three percent of respondents favoured short courses with “hands-on” practice. Eighty-nine percent agreed that these courses should be run and coordinated by the Health Sciences Research Office instead of the departments. There was use of varied statistical packages requiring one package for standardised support. The numbers of postgraduate students attending short courses in statistics increased from 2010 to 2012 as did the numbers attending statistical consultations. Graduations and publication outputs increased over this period of time although this may not be directly linked solely to the biostatistical support system introduced.

**Conclusions:**

There is a distinct need for biostatistics training in developing countries and the process described in this study could be replicated in any health sciences institution, especially in a resource-restricted environment.

## Background

Health research plays a key role in strengthening health and solving health needs. As Kellerman *et al.* [1] noted “the capacity to produce relevant research has been identified as a limitation to improved population health”. This process should not be undervalued in Africa where many countries suffer from the burden of multiple diseases. However, in order to conduct research relevant to population needs, it is necessary to have some comprehension of biostatistics. It has been shown that even in developed countries most medical residents lack the level of knowledge of biostatistics required to understand the results published in clinical research [2]. In order to address and support biostatistics for health research, the Health Sciences Research Office of the University of the Witwatersrand sought to introduce training in biostatistics to sustain research and postgraduate education.

Biostatistics has become an integral part of health research for the design, management and analysis of data arising from studies in clinical and non-clinical research, epidemiology and social sciences. Such “statistically expanded” research, especially in the health sciences arena, is conducted by academic members of staff and postgraduate students. As the use, and understanding, of biostatistics is essential for researchers, increasing training would enhance support in protocol development and study design, data collection and statistical analysis. Recently, both Macleod *et al.* [3] and Ioannidis *et al.* [4] highlighted problems leading to weaknesses in design, conduct and analysis of biomedical and public health research.

Further, biostatistics training and support is central to clinical decision making and also health management [5]. In a study conducted by West and Ficalora [6] at an institution in the US, the majority of clinicians who responded believed that a better understanding of biostatistics would benefit their careers.

Capacity to provide biostatistics support in developing countries is relatively lacking and thus, developing countries look to developed countries for standards of statistical practice and training [7–10]. A workshop conducted in Bethesda, Maryland in 2009 on strengthening biostatistics resources in sub-Saharan Africa (SSA) was followed up by a second workshop in Botswana in 2011 [10]. The workshops recognised the need for enhanced biostatistics capacity in SSA in order to improve biostatistics support to the increasing and emerging biomedical research being conducted in academic and research institutions. Coupled with insufficient training in biostatistics in the SSA region [10], there is limited biostatistics support to enhance research output from academic institutions in these countries. Training should be provided for clinicians and researchers early in the planning of their research in order to avoid errors at the design stage of their studies which are difficult to rectify once a study has been undertaken [11].

While distinct structures for the teaching and support of biostatistics may exist in developed countries, there is little published information in the literature [12]. One example is that of the University of Pennsylvania, Perelman School of Clinical Medicine where a Centre for Clinical Epidemiology and Biostatistics exists, which trains individuals and supports research projects in clinical research [13]. In Australia, while none of the medical schools audited fully integrated the teaching of statistics into their problem-based learning courses, statistics courses were provided in different formats [14]. While standard biostatistics courses focused on Masters and PhDs are ongoing, teaching in US medical and health-related institutions is going the online route [15]. The requirement for biostatistical training has been acknowledged in a number of developing countries such as Vietnam [16], Pakistan [17], India [5, 11] and Turkey [18] and as this is indicated in publications, is perceived to be of greater need with respect to health sciences training in these countries .

Limited literature on biostatistical training could be found for the African region [8, 10]. In Africa, although there are no known institutions which provide institutional-wide biostatistics research support, the funding of, and investment in, statistics is increasing [19]. Biostatistical support is said to be hindered by high teaching loads relative to the available human capacity and resources (personal communication).

Although human and financial resources are limited, the University of the Witwatersrand Faculty of Health Sciences (Wits FHS) recognized the need for institutional support for biostatistics and other courses required to enrich research. Resources were committed to strengthen research, improve postgraduate throughput and enhance skills in statistical analysis and understanding. This study provides the strategy to identify the need and gaps for biostatistcal support. It then further discusses creation, operation, financial costs and outcomes in setting up such statistical support in a limited resource, developing country.

## Methods

### Study setting

Located in Johannesburg, Gauteng province, Wits is an urban, comprehensive university which has a distinctive capacity to contribute to the reconstruction and development of South Africa and the region, through research and the production of skilled graduates The FHS consists of seven Schools (see Table 1) where academic activities include under- and post-graduate teaching and research. Two of the authors (TC and EL) are in charge of conducting biostatistical courses for the Faculty of Health Sciences and supervising the tutors in charge of the statistical consultations. The third author (BK) is responsible for directing all postgraduate and research activities in the Faculty of Health Sciences.

**Table 1:** Frequency distribution and biostatistics needs of respondents to questions (in bold). (Needs Assessment survey, University of the Witwatersrand, Faculty of Health Sciences, 2009)

Characteristic	Total (*n* = 110)
**What is your School?**	
Anatomical Sciences (%)	2 (1.9)
Clinical Medicine (%)	46 (43.0)
Oral Health Sciences (%)	4 (3.7)
Pathology (%)	15 (14.0)
Physiology (%)	8 (7.5)
Public Health (%)	14 (12.2)
Therapeutic Sciences (%)	19 (17.8)
**Are you able to analyze data?**	
Yes (%)	74 (67.3)
No (%)	36 (32.7)
**Should biostatistics be taught to postgraduate students and staff?**	
Yes (%)	98 (89.1)
No (%)	1 (0.9)
Don’t know (%)	11 (10.0)
**Would you be willing to teach on biostatistics courses?**	
Yes (%)	27 (24.6)
No (%)	83 (75.4)
**At what level would you be willing to teach courses?** ^**a**^	
Basic (%)	23/27 (85.2)
Intermediate (%)	8/27 (29.6)
Advanced (%)	-
**Which arrangement for biostatistics support do you prefer?** ^**a**^	
Group consultation at specified times (%)	57 (51.8)
Short courses with hands-on support (%)	80 (72.7)
Statistical co-supervision (%)	56 (50.9)
Other arrangements (%)	5 (4.5)
**Which times for biostatistics short courses do you prefer?**	
During office Hours (%)	58 (80.6)
During weekends (%)	6 (8.3)
After working hours (%)	8 (11.1)
Other times (%)	-
**What statistical packages are you currently using?** ^**a**^	
EPI INFO (%)	31 (28.2)
STATA (%)	22 (20.0)
STATISTICA (%)	19 (17.3)
SAS (%)	14 (12.7)
SPSS (%)	18 (16.4)
Other Packages (%)	15 (14.2)
**Would you be willing to use STATA?**	
Yes (%)	68 (63.0)
No (%)	10 (9.3)
Don’t Know (%)	30 (27.8)
**Do you require training in STATA?**	
Yes (%)	82 (75.2)
No (%)	15 (13.8)
Don’t Know (%)	12 (11.0)

^a^Multiple responses allowed

### Study design

Sporadic disjointed biostatistical courses at Wits were introduced in 2008. This cross-sectional study was initiated to ensure a well-publicised and coordinated statistical support at FHS level. To ensure that this was operationalised, the study was designed and conducted in two phases.

### Phase one: strategy and creation of support

The first phase, conducted in February 2009, was an anonymous cross sectional survey investigating statistical needs within the FHS. Based on the identified gaps and needs from this survey, creation of a support system which included short courses in biostatistics, topical biostatistics seminars, walk-in and booked one-on-one statistical consultations with senior biostatisticians of the FHS were offered. The one-on-one consultations were referrals from the walk-in consultations with tutors. These tutors were either MSc students who performed well on biostatistics coursework on the Epidemiology programme within the School of Public Health or PhD students with skills in quantitative analysis. The short courses targeted both staff and postgraduate students.

### Phase two: operation and outcomes

Having conducted a needs assessment survey and created a biostatistics support system within health sciences, it was essential to monitor the operations of the system on an on-going basis. As such, the second anonymous cross-sectional survey was conducted between January 2010 and December 2011. The aim of this survey was to document feedback from institutional staff and postgraduate students, specifically on statistical support provided during the walk-in consultations as a first point of contact. Feedback from this survey was an important process outcome of the system as it helped to assess and review procedures.

To complement these surveys, an additional outcome included the numbers of postgraduate students completing their studies since the introduction of the statistical support. The postgraduate degrees reviewed included the Masters by coursework (specific to the field in which the degree is being undertaken) and research (only 30 % contributed by a research project to the degree), the Masters by research (100 % research) and Ph.Ds. In addition, the number of publications generated during this period was also calculated. The annual financial budget and human resources utilised to provide the service were compiled, including the financial costs of the biostatistical packages used.

### Sample size

In the first survey, the intention was to estimate the proportion of individuals who needed biostatistics support. No known studies have been conducted to estimate this in African academic institutions. In order to estimate the required sample size we assumed a value of 50 %. In this regard, if the need for biostatistics support was estimated to within 10 % precision at 95 % confidence level, we would require a minimum of 110 participants in the survey. This takes into account a 10 % non-response rate.

Further, assuming that 70 % of the FHS postgraduate students are truly satisfied with the biostatistics support provided, a minimum of 126 participants who attended the statistical consultations was required to determine levels of satisfaction to within 8 % precision and at 95 % confidence level.

### Data collection

For the needs assessment survey, self-administered questionnaires were circulated to FHS academic members of staff (approximately 1300) whose names and contacts appeared on the staff list. These questionnaires were sent through email addresses by the Research Coordinator and follow-ups were made until the required minimum sample size was attained. There was no relationship between the administrator of the survey and the biostatisticians.

For the second survey on feedback, the information that was collected from FHS staff and postgraduate students who attended the consultations included the type of support sought, how they rated the consultation, as well as the strengths and weaknesses of the support service. These questionnaires were filled in as an exit interview to the walk-in consultation. Closed questions were utilized for both surveys (with an option to add comments). Some questions were open-ended. Answers were classified according to similarity. Repeat consultations were considered as a separate visit and were evaluated independently. The feedback questionnaire forms were provided to the individuals by the tutor and once completed, were placed in a sealed box outside the consultation room. The questionnaire was anonymous.

### Courses and walk-in consultations

The teaching of the courses was conducted by three biostatisticians each of whom had a PhD qualification. These biostatisticians were in other Institutional posts, but contributed 20 % of their time to the biostatistical training.

The walk-in consultations were offered by MSc students or PhD students from the Epidemiology and Biostatistics programme in the School of Public Health with quantitative skills (collectively referred to as tutors). Consultations were provided on three specific days of the week. A total of nine hours per week were ring-fenced for one-on-one consultations. There is a register of attendance for short courses and for consultations. A register and an exit interview are also kept in the HSRO. These are filed for record purposes to show uptake. Two institutionally approved statistical software packages (STATA and STATISTICA) were used.

### Financial costs

The total cost of the senior biostatisticians who organized and delivered the teaching in the unit (calculated as 20 % per statistician for the time provided) was the equivalent of USD 42 000. This amount did not include the time spent on preparation of the lectures for the biostatisticians due to financial constraints. These senior biostatisticians who were in funded posts, were not paid additionally for the biostatistics support which they provided in the Research Office. Additional funding of USD 5 000 per annum for 3 years was provided by the central University and was used for the appointment of three part-time tutors. The latter funding was increased to USD10 000 in 2012.

Statistical packages (STATA and Statistica) were bought at a cost of USD 16 000 for staff and student licenses, as well as a number of individual licenses. As the use of one package increased, the fee for the license was slightly reduced. In addition, Wits has access to SAS (SAS Institute Inc., Cary, NC, USA) and Epi-Info™ for use on the general Institutional network.

Thus, the total cost in relation to biostatistics support in the Faculty of Health Sciences for 1 year was USD 63 000. The cost to the institution per hour in relation to the formal courses and consultations was approximately USD 70. This cost is reasonably high in an academic setting, but below average for industry in South Africa (personal communication). These costs do not include computers and infrastructure. In 2012 the total cost increased to just over USD 68 000.

### Data management and analysis

Data was entered in Epi-Info version 3.5.1 twice and was transferred to STATA version 13 for analysis. Data management included checking for missing data, range and consistency checks. For any errors, this was checked back to the hard copy questionnaires and corrected where data was available or incorrectly entered. Programme files to check and correct for errors were created.

The analysis for this study was mainly descriptive. Similar open-ended questions were grouped into a category and the percentage of each category was calculated against the total number of answers. Frequency tables of factors such as school, preferred arrangement for biostatistics support and levels of biostatistics knowledge were provided as frequencies with corresponding percentages. Ninety five percent confidence intervals (95 % CI) were calculated for the key outcomes of the needs assessment survey.

Authorization for use of the data for this study was obtained from the Human Research Ethics Committee (Medical), University of the Witwatersrand, Johannesburg (M1211112), with a recommendation for anonymization of data. Thus gender and age of the individual staff and postgraduate students were not collected.

## Results

### Needs assessment survey

One hundred and ten academic members of staff responded to the needs assessment survey (Phase One). Table 1 shows a frequency distribution of the characteristics of these staff members. Forty-three percent (46) of them were from the School of Clinical Medicine, 17.8 % (19) from the School of Therapeutic Sciences Pathology (14 %) and 12.2 % from the School of Public Health. Representation from the remaining schools ranged from 1.9 to 7.5 %.

The Needs Assessment Survey has shown that 32.7 % (95 % CI: 24.1–42.3 %) of academic staff members were not able to analyse their own data, and 89.1 % (95 % CI: 81.7–94.2 %) agreed that biostatistics courses should be offered. Thirty-six percent (27/75) of the staff who were able to do their own analysis agreed to teach on proposed institutional-wide biostatistics short courses, with 85.2 % (23/27) and 29.6 % (8/27) willing to do so at basic and intermediate level of statistics short courses respectively.

About 72.7 % of staff chose biostatistics short course training with “hands-on” practice; 51.8 % selected group statistical consultations at specified times and 50.9 % opted for biostatistician co-supervision of postgraduate students. Further, 80.6 % (58) of staff chose short courses that run specifically for postgraduate students to be conducted during working hours compared to weekends (8.3 %) and after working hours (11 %).

The reported use of statistical packages varied. These included those packages provided by the Faculty of Health Sciences such as STATISTICA, StatSoft® (Tulsa, OK, USA); STATA® (StataCorp LP, College Station, Texas, USA; or those from Wits as an institution (SAS, SAS Institute Inc., Cary, NC, USA) or downloaded free (Epi-Info™). Epi-Info was popular and used by 28.2 % (31) of the respondents followed by STATA (20 %).

Table 2 shows frequencies of statistical topics commonly chosen by both staff and postgraduate students. Some of the staff members were also registered for higher degree qualifications. The five most favoured topics included data description, hypothesis testing, correlation and linear regression, sample size and logistic regression. The basic topics, data description (79.6 % vs 74.5 %) and hypothesis testing (81.6 % vs 77.6 %) were more preferred by postgraduate students than staff. The intermediate topics, correlation and linear regression (59.2 vs 73.5 %) and logistic regression (41.8 % vs 62.2 %) were preferred more by staff than postgraduate students. The topics listed were categorised into basic statistics courses and advanced statistics, with the basic consisting of data description, hypothesis testing, correlation and linear regression. The advanced short courses consisted of sample size, logistic, survival and longitudinal data analysis.

**Table 2:** Frequency distribution of topics suggested by staff and postgraduate students (a Needs Assessment Survey, University of the Witwatersrand, Faculty of Health Sciences, 2009)*

	Total (*n* = 110)	
Statistics Topics	For staff	Students
Data Description (%)	73 (74 · 5)	78 (79 · 6)
Hypothesis Testing (%)	76 (77 · 6)	80 (81 · 6)
Correlation and Linear Regression (%)	72 (73 · 5)	58 (59 · 2)
Logistic Regression (%)	61 (62 · 2)	41 (41 · 8)
Survival Analysis (%)	40 (40 · 8)	24 (24 · 5)
Poisson Regression (%)	35 (35 · 7)	23 (23 · 5)
Longitudinal Data Analysis (%)	48 (49 · 0)	35 (35 · 7)
Clinical Trials (%)	48 (49 · 0)	33 (33 · 7)
Sample Size Calculation (%)	71 (72 · 5)	61 (62 · 2)

*multiples answers allowed

### Courses

**Fig. 1:**
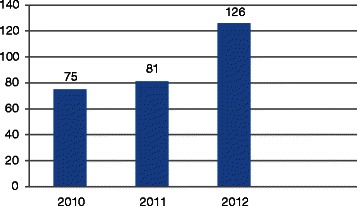
Frequency tabulations of students’ attendance of biostatistics short course from 2010 to 2012, University of the Witwatersrand, Faculty of Health Sciences

Implementation of extra statistical basic courses and the addition of an advanced statistics course started in 2009. Basic statistics courses of 20 h duration were offered and repeated three times a year; with the advanced statistics course delivered at other times of the year. All courses were split between didactic lectures (40 %) and practical work (60 %). No formal assessment of attendees was undertaken. The numbers of postgraduate students attending these courses increased from 75 in 2010 to 126 in 2012 as is depicted in Fig. 1. Attendance for each of the courses ranged between 10 and 15 participants.

### Consultations

The uptake of statistical consultation has been increasing over the years. Prior to 2010, only one-on-one consultations were being conducted by a senior biostatistician. With apparent increasing uptake, walk-in consultations between a tutor and a postgraduate student were initiated in 2010 parallel to one-on-one consultations. The number of hours per year for all types of consultation increased from 2010 to 2012. For example, there were 571 h of consultations in 2010 compared to 662 and 714 h in 2011 and 2012 respectively. This is in addition to 726 h a year since 2010 for booked referral consultations.

### Feedback on consultations

Table 3 shows the frequency distribution of feedback from postgraduate students (exit interview) on walk-in consultations, including strengths and weaknesses of this support system. The responses to questions in Table 3 were fixed but with an “Other” option for individuals who have different suggestions than the answers provided. Responses to questions on strengths and weaknesses of the support service were open-ended.

**Table 3:** Feedback from staff and postgraduate students on individual walk-in consultations (University of the Witwatersrand, Faculty of Health Sciences 2010–2012)

Factors of interest	Total (*n* = 167)
Type of client who came for consultation	
Staff (%)	31 (18.9)
Postgraduate Students (%)	111 (67.7)
Both staff and postgraduate student (%)	22 (13.4)
Did the consultation help you with your research project	
Excellent (%)	104 (62.7)
Very Good (%)	44 (26.5)
Good (%)	18 (10.8)
Bad	
Very bad	
Were any new skills learnt during the consultation?	
Yes (%)	151 (90.4)
No (%)	5 (3.0)
Not sure (%)	11 (6.6)
Would you recommend the service?	
Yes (%)	160 (95.8)
No (%)	3 (1.8)
Not sure (%)	4 (2.4)
Open question - Strengths of the support service (*n* = 134)	
Presence of Senior Support Staff (%)	13 (9.7)
Helpful with good explanations (%)	55 (41.0)
Good clarification of stats questions (%)	30 (22.4)
Professional knowledge of statistics (%)	23 (17.2)
Sufficient time for one-on-one session (%)	6 (4.5)
Other strengths mentioned (%)	7 (5.2)
Open question - Weaknesses of the support service (*n* = 39)	
Not Enough consultants (%)	6 (15.4)
Not enough time (2–4 pm) (%)	16 (41.0)
Poor booking times (%)	2 (5.1)
Limited resources for consultation (%)	2 (5.1)
Any other weaknesses (%)	13 (33.3)

A total of 167 staff and postgraduate students filled in the questionnaires at the end of a consultation. The denominators in the table vary due to non-response to some questions. The majority of the respondents (81.1 %) were either postgraduate students or staff members who were also registered for postgraduate studies in the Institution.

Among those who sought support through the consultations, 62.7 % (104) indicated that the consultation had helped with their research project, with 90.4 % (126) reporting that they had learnt new skills. The survey shows that 95.8 % (160) would recommend the service to other postgraduate students and staff.

There were varied reasons given as strengths for the walk-in consultations, as shown in Table 3. The majority of students (41 %) found the consultations very helpful and good explanations to their problems were provided. The reported weaknesses of this statistical support included limited time for consultations (16/39 = 41.0 %), and insufficient numbers of biostatistics tutors to handle the large number of students (6/39 = 15.0 %) accessing the service. Thirty three percent reported “other weaknesses” (*n* = 13) which included the need for a “better ventilation system”, “the support service was not advertised well”, “not enough space”, “time of day for consultations”, “location”, and “different statistical packages used”.

**Fig. 2:**
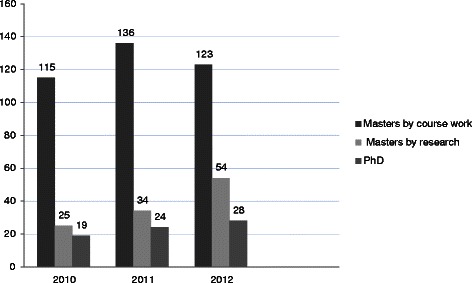
Number of graduates sorted according to the type of postgraduate degree: 2010–2012, University of the Witwatersrand, Faculty of Health Sciences

An increase in the number of graduations of Masters by dissertation and of PhDs is evident in Fig. 2. The Masters by dissertation graduations elevated to 54 in 2012 by comparison with 25 in 2010. Marked increases in the graduations of student groups occurred in 2013 (MSc by coursework and research 163; MSc by dissertation 43; PhD 45) following the first graduation of the year) (not shown in Fig. 2) although such an increase cannot be fully attributed to biostatistics support alone.

### Publication outputs

The total publications produced by staff over the years 2010 to 2012 are depicted in Table 4. The publication numbers increased from 482 in 2010 to 813 in 2012 and may be indirectly linked to the inception of the biostatistical courses.

**Table 4:** Publication outputs for the Faculty of Health Sciences, University of the Witwatersrand between 2010 and 2012

Publication Year	Total publications^a^
2010	482
2011	799
2012	813

^a^Total publications include journal articles, chapters in books, book conference proceedings and reviews

## Discussion

While the minimum sample size was attained in our survey, we had expected a much larger response from staff. Not all the individuals who participated in workshops and consultations completed the evaluation forms. Thus, there is potential for bias as only a subset of individuals might have responded to both surveys. However, for purposes of setting up a system, we found the responses quite informative and these confirmed views on the needs of such support structures.

The ‘needs analysis’ showed that short courses with hands-on practice in biostatistics were most favoured for research and that training should be provided during working hours. This finding of ‘biostatistics training and support need’ is similar to that described by Gore *et al.* [5], who not only described this “need” by academics and postgraduate students in India, but also the wish of these academics to upgrade their knowledge of biostastistics with refresher training programmes.

Both structured courses and support through one-on-one consultations were requested and were implemented in our Institution. Unlike Singh *et al.* [11] who undertook a situational analysis on biostatistics education in India, we were unable to determine the nature and number of biostatistics courses throughout Africa. While the South African Medical Research Council offers support to postgraduate students and researchers and research support is also available at the University of Malawi (http://www.rsc.medcol.mw), other statistical support directed towards health sciences may exist in the Southern African region, but has not been documented to our knowledge. Singh *et al.* [11] found that only 19 institutions in India offer various courses in biostatistics or biometry. Of the 19 institutions, the majority provided full time courses of one year’s duration. One-on-one consultations were not mentioned in the latter paper.

Although only few respondents (4 · 5 %) in our study commented on the need for one-on-one consultations, this was set in place and is becoming more sought after. The strategy behind this decision was that short courses which were offered should be complemented/followed up by consultations. It was felt that group consultations would not work because of the varied and unique statistical problems which students have. Therefore, short courses for groups were preferred followed by on-going consultations (walk-in or booked one-on-one). At our Institution, Libhaber and Vorster [20] observed that between 8 and 18 consultations per month were occurring (which translates into approximately 72–162 h p.a. per consultant). Considering the high demand for the consultations, the service is now offered on 12 days a month.

The increase in the attendance at courses at our Institution over the last number of years indicates the need for training in biostatistics. Because of the key role South African institutions play in capacity building in the region [1], it is important that biostatistical training is increased. This will ensure sustained regional contributions to medical research and development. However, one of the limitations to biostatistics training which is encountered in our Institution is that due to high clinical service provision, clinicians who are also studying for their postgraduate degrees find it difficult to fit such training into working hours. Because of the quadruple burden of disease in the region, the shortage of staff and high patient-doctor ratios, clinicians have high workloads at health facilities and work long hours. This is supported by the findings of Dodani and LaPorte [17] who suggest flexibility in terms of content, form and outcome of these courses.

For the future, due to the paucity of trained biostatisticians available to undertake such support, it is envisaged that some of our courses should be made available online. The introduction of online courses in research ethics and biostatistics were as useful in upgrading the knowledge of Indian scientists who undertook these, as were the “traditional” on-site courses [21].

In a resource-restricted environment such as that in our Institution, it has been important to set up support for research training, including that in biostatistics, on a very limited budget. As is illustrated in this study it was possible to set up biostatistical support at a cost of approximately US $68 000 per year. Statistics services are expensive [13], but at the costs described in the present study, should be affordable to all institutions, not only those in resource-restricted environments. While we do not foresee that the introduction of online courses will reduce the costs of supporting biostatistics in the Faculty of Health Sciences, it will enable those of our staff and students based off-site to engage with the courses and improve their knowledge. The setting up of biostatistics courses and consultations in-house is of financial benefit to staff and students as in the US the cost of an individual statistics consultation is $75 per hour with a Masters level statistician and $150 with a senior/doctoral level statistician [22]. In the present study, the cost is low in relation to local industry.

The investment in the setting up of this system was certainly of benefit to the Institution as is evidenced by the increased publication output and graduation of students. While the introduction of a formal biostatistics system may not have been the sole reason for this increase, they may be due to an ecological association related to the increased uptake of courses. Other initiatives which may have contributed to these increases are writing retreats, research methodology and protocol development workshops.

Though some academic members of staff (24 · 6 %) expressed willingness to teach biostatistics, due to other teaching commitments none actually came forward to assist with this teaching. Further, limited funding is available to incentivize staff to participate as teachers of biostatistics. A potential source for the teaching of basic biostatistics is a pool of postgraduate students currently studying for an MSc in Epidemiology and Biostatistics within the Institution. These students could also act as tutors for one-on-one consultations. However, due to financial constraints, the Institution is not being able to employ the tutors in a permanent post. Therefore they may seek other job opportunities outside our institution. Even in developed countries the demand for biostatisticians far exceeds the supply [8].

As an institution in a developing country, the importance of the expansion of research skills such as biostatistics, scientific writing, and grant writing amongst others, as part of building local and regional research capacity is imperative. This brief on increasing research capacity in developing countries is echoed by many authors [1, 23–25] and will lead to an expansion of capabilities in order to improve the health needs of these countries.

## Conclusion

A distinct need for biostatistics training was identified in our Faculty of Health Sciences. Both structured courses and support which included group consultations and statistical co-supervision and one-on-one consultations were requested. An increase in biostatistical course attendance occurred from 2008 to 2012. Increases in the number of postgraduate students graduating with a Masters by Research or a PhD were seen from 2010. Publication numbers also showed a slight increase from 2010 although we have expressed caution in viewing such increases as directly linked to biostatistics courses and consultations. The financial costs for the setting up of a training and support system are not prohibitive.

Based on these experiences in our Institution, the system described in this study could similarly be set up in any health sciences facility or school, especially those which are under-resourced. In the near future, the Faculty of Health Sciences is working on making a basic statistics course compulsory for all its postgraduate students. In a resource-restricted environment such as that in southern Africa it would be of benefit to collaborate with our African counterparts on extending our current support systems in biostatistics.
